# Inter-Simple Sequence Repeat Data Reveals High Genetic Diversity in Wild Populations of the Narrowly Distributed Endemic *Lilium regale* in the Minjiang River Valley of China

**DOI:** 10.1371/journal.pone.0118831

**Published:** 2015-03-23

**Authors:** Zhu-hua Wu, Jisen Shi, Meng-li Xi, Fu-xing Jiang, Ming-wen Deng, Selvadurai Dayanandan

**Affiliations:** 1 College of Landscape Architecture of Nanjing Forestry University, Nanjing, China; 2 Key Laboratory of Forest Genetics and Biotechnology, Ministry of Education, College of Forestry, Nanjing Forestry University, Nanjing, China; 3 Forest and Evolutionary Genomics Laboratory and Centre for Structural and Functional Genomics, Biology Department, Concordia University, Montreal, Quebec, Canada; 4 Quebec Centre for Biodiversity Sciences, Montreal, Quebec, Canada; United States Department of Agriculture, UNITED STATES

## Abstract

*Lilium regale* E.H. Wilson is endemic to a narrow geographic area in the Minjiang River valley in southwestern China, and is considered an important germplasm for breeding commercially valuable lily varieties, due to its vigorous growth, resistance to diseases and tolerance for low moisture. We analyzed the genetic diversity of eight populations of *L*. *regale* sampled across the entire natural distribution range of the species using Inter-Simple Sequence Repeat markers. The genetic diversity (expected heterozygosity= 0.3356) was higher than those reported for other narrowly distributed endemic plants. The levels of inbreeding (*F*_st_ = 0.1897) were low, and most of the genetic variability was found to be within (80.91%) than amongpopulations (19.09%). An indirect estimate of historical levels of gene flow (*N*_m_ =1.0678) indicated high levels of gene flow among populations. The eight analyzed populations clustered into three genetically distinct groups. Based on these results, we recommend conservation of large populations representing these three genetically distinct groups.

## Introduction

Lilies (*Lilium* spp.) are perennial herbaceous monocots of the family Liliaceae and are commercially important as ornamental plants throughout the world. In addition, lily bulbs are regularly consumed as food in Asia [1], and some species, including *L*. *longiflorum* Thunb., *L*.*brownii*F.E.Br. ex Miellez var. *viridulum* Baker, *L*. *pensylvanicum* Ker Gawl. And *L*. *pumilum* DC, have been used traditionally in China as sedative, anti-inflammatory and antitussive agents(cough suppressants)[2,3]. The crude extract “Baihe” is prepared from the bulbs of *Lilium* spp. and is regularly used to treat lung ailments in China [4].

The native range of *Lilium* is centered around China with almost half of the species being distributed within China [5].*Lilium regale* Wilson is endemic to southwestern China, with the distribution limited to an area of approximately170 km^2^ in the valley region within the upper reach of the Minjiang River [6,7].*L*. *regale* blooms annually from May through June, with conspicuous white trumpet-shaped flowers. Since the introduction of *L*. *regale* to North America during the period from 1907 to 1911 by Wilson [8],due to its vigorous growth and resistance to diseases[9,10,11], it has been widely used as a parent in breeding programs and this has led to the production of many hybrid cultivars [12],including some resistant to the devastating wilt disease of lilies [8].

The native habitats of *L*. *regale* are subject to severe anthropogenic and natural disturbances including land use changes, landslides and earthquakes [13], which threaten the survival of the species. As *L*. *regale* is a commercially important endemic species restricted to a narrow geographic area in which severe environmental disturbances are likely to occur, conservation and management plans to protect wild populations are urgently needed. The long-term survival of the species depends on the maintenance of sufficient genetic variability to respond and adapt to new selective pressures under changing environmental conditions [14,15,16]. As such, the assessment of genetic variability serves as the first step for evaluating the long-term survival potential of a given species and developing suitable conservation programs [17,18,19].

In general, species with a narrow range of distribution are considered to have lower levels of genetic diversity than their more widespread counterparts, as they undergo genetic drift, inbreeding and low levels of gene flow resulting from historically small and fragmented populations [20]. Although some studies support this view[21,22,23,24], others have revealed moderate to high levels of genetic diversity in some endemic species with narrow distributional ranges [16,25,26]. Life history traits such as the mating system, seed and pollen dispersal strategies, and mode of reproduction also influence genetic diversity within and among populations[19,20,27].

To date, the genetic diversity of only six *Lilium* species has been assessed using molecular techniques[28,29,30,31,32,33]. Random Amplified Polymorphic DNA markers have been used to assess the genetic variation in Turk's-cap lily (*L*. *martugon* L.), one of the most common lily species in Europe [28], the Taiwan lily (*L*. *longiflorum* Thunb. var. *formosanum* Baker)distributed in wide altitudinal ranges from the lowlands to high mountains of Taiwan[29], and *L*. *brownii* in southern China[30]. A chloroplast DNA and Inter-Simple Sequence Repeat (ISSR) marker-based study evaluated the genetic diversity of *L*.*maculatum* var. *bukosanense*, the Miyamasukashi-yuri lily, which is endemic to Japan[31].Simple Sequence Repeat (SSR)-based studies have focused on characterizing the genetic diversity and structure of *L*. *philadelphicum* in the midwestern United States [32]and of *L*. *japonicum* Thunb. var. *Abeanum*(Honda) Kitam, one of the rarest plants in Japan [33].

In the present study, we used Inter Simple Sequence Repeat (ISSR) data to analyze genetic diversity of *L*. *regale* populations across its entire natural range. ISSR markers represent mostly noncoding regions of the genome flanked by SSRs. Several genetic studies of natural populations using ISSR markers have demonstrated their high variability, reproducibility [16,34,35,36] and relative ease of use in population-level studies in a variety of organisms [36,37,38] including plants [15,22,39,40,41]. ISSR markers are particularly useful for population genetic studies of rare species for which no previously characterized molecular markers are available [21,22,40,41]. We analyzed eight populations of *L*. *regale* distributed in the Minjiang River valley of China using ISSR markers to assess the levels and distribution of genetic diversity within and among natural populations.

## Materials and Methods

### Sample collection and sampling sites

The Minjiang River valley is relatively dry compared to humid surroundings in southwestern China, making this a unique habitat for plants. *L*.*Regale* is not an endangered or protected species and therefore no specific permission is currently required for academic studies. Although *L*. *regale* individuals are abundant in the area, they are not prominent because of their small size relative to the dominant vegetation, which consists of several shrub species including *Caryopteris incana*,*Ceratostigma minus*,*Lespedeza Formosa* and *Artemisia annua*[7].Therefore, we collected samples during the flowering season in May and June, when *L*. *regale* plants can be easily detected.

**Fig 1:**
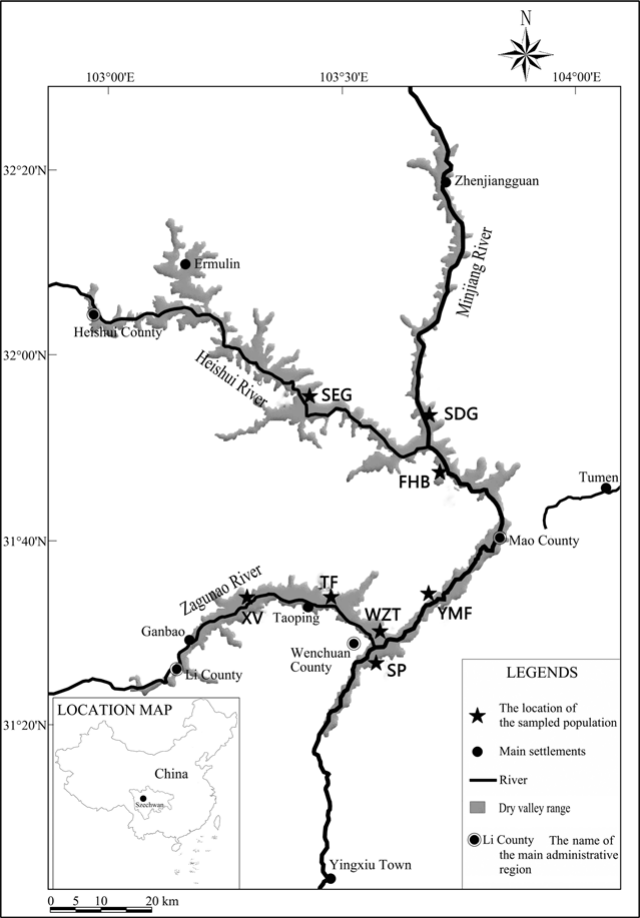
Locations of *L*. *regale* sampling sites along the Minjiang River valley. Population names are listed in Table 1.

We first surveyed for *L*.*regale* within its established distribution region [5,7,8] by direct searching and consulting with local forest managers and farmers. We determined the approximate current distribution (Fig. 1), which comprised three major rivers(Minjiang, Heishui, and Zagunao)within the Minjiang valley. Then, based on the potential impact of the watershed on genetic diversity and accessibility of populations, eight sites were chosen for sampling, including five sites along both banks of the Minjiang River, one site in the Heishui tributary valley, and two sites in the Zagunao tributary valley(Table 1, Fig. 1).

**Table 1:** Locations and sample sizes of 8 populations of *L*. *regale* used in the present study.

Population	Site	Valley	Longitude, and latitude	Altitude (m)	Location relative to river	Location on the slope	Sampling number
FHB	Feihong Bridge, Mao County	Minjiang River	103°45′31″E,31°48′26″N	2 200	west	middle	23
SDG	Shida Gate, Mao County	Minjiang River	103°43′10″E,31°52′22″N	1 770	west	middle to upper	20
SEG	Seergu, Heishui County	Heishui River	103°25′5″E, 31°54′47″N	1 820	east	lower to middle	23
SP	Sha Pit, Wenchuan County	Minjiang River	103°36′32″E,31°28′43″N	1 600	west	middle	23
TF	Tao Flat, Li County	Zagunao River	103°29′47″E, 31°35′29″N	1 500	north	middle	21
WZT	Weizhou Town, Wenchuan County	Zagunao River	103°37′45″E,31°30′35″N	1 700	east	middle to upper	19
XV	Xue Village, Li County	Zagunao River	103°20′47″E, 31°32′26″N	1 630	south	middle	23
YMF	Yangmao Flat, Lanxin Town, Mao County	Minjiang River	103°44′17″E,31°35′12″N	1 500–1 800	east	middle	20

Leaf samples from19–23 individuals [42]located at 2-m intervals along a transect in each site were collected, placed in sealed plastic bags containing silica gel to prevent degradation of DNA, transported to the laboratory, and stored at -80°C until extraction of the genomic DNA.

### DNA extraction and ISSR amplification

The leaf tissue (100mg) from each sampled individual was ground to powder in liquid nitrogen, and total genomic DNA was extracted following the CTAB-based procedure of Armaleo et al.[43]. The extracted DNA was amplified by PCR using 100 ISSR primers obtained from the University of British Columbia and 35 *Lilium*-specific ISSR primers from Masumi Yamagishi[44],which were synthesized by SBSGenetechCo., Ltd.Only 10 primers produced clear and reproducible bands and were selected for subsequent genotyping (Table 2).

**Table 2:** The ISSR primer sequences, number of bands observed and assessment of banding patterns.

Primer	5′–3′ sequence	Number of amplified bands	Number of polymorphic loci(EMR)	Proportion of polymorphic loci(%)	the polymorphic information content (PIC)	the marker index(MI)	Shannon’sIndex(*H*′)
3A01	(GA)_8_TC	19	19	100	0.2041	3.8779	0.3171
3A21	(CA)_7_GTA	22	21	95.4	0.2071	4.3491	0.3351
3A42	(GACA)_4_C	18	17	94	0.1597	2.7149	0.2779
3A54	(AG)8GT	18	18	100	0.2458	4.4244	0.3758
3A62	(TG)_7_ACT	16	15	93.7	0.1703	2.5545	0.2865
3A07	(TG)_7_ACC	17	17	100	0.2232	3.7944	0.3858
3A39	(AG)_7_CTT	20	20	100	0.2783	5.566	0.3526
3A53	(AG)8CA	10	9	90	0.2332	2.0988	0.3452
3A56	(TG)_7_ACG	19	19	100	0.2394	4.5486	0.3719
UBC873	(GACA)4	21	21	100	0.194	4.074	0.3237
average		18	17.6	97.3	0.21551	3.80026	0.3387

The genotyping of PCR reactions was performed in 20-μl reaction volumes containing 20 ngtemplate DNA,0.4mMdNTPs, 1.25 mM MgCl_2_,0.6μM of each primer, 0.5U*Taq*-polymerase(Takara),2μl of 10×PCR buffer and double-distilled water. The thermal cycling steps consisted of an initial denaturation at 94°C for 5 minutes, followed by 45 cycles of denaturation at 94°C for 40 seconds, annealing at 52°C for 45 seconds and extension at 72°C for 90 seconds, followed by a final extension at 72°C for 8 minutes. PCR amplification was performed in a PTC-200 DNA thermocycler (MJ Research, Boston, MA). The amplification products were separated on 1.5% agarose gels containing 0.5 mg/ml ethidium bromide in 1× Tris-borate-EDTA buffer. The gels were examined under ultraviolet light and photographed using a Kodak EDAS 200 digital documentation system. All samples were analyzed at least twice with each primer to assess the reproducibility of ISSR-based genotyping.

### Data analysis

The ISSR banding pattern of each individual was scored as the presence or absence of a given band to construct a binary data matrix (1 and 0) consisting of 172 individuals. The informativeness of markers and diversity was evaluated using marker index (MI) [45], Shannon’s index (*H*′) [46] and the polymorphic information content(PIC) [47,48]. The PIC of dominant bi-allelic data was estimated by the formula PIC = 1-*p*_i_^2^-*q*_i_^2^, where *p* is the frequency of visual alleles and *q* is the frequency of null alleles (Hardy-Weinberg equilibrium was assumed, where *q* = (1band frequency)/2 and *p* = 1-*q*).MI was calculated as MI = PIC × number of polymorphic loci. Shannon’s index (*H*′) was calculated by the formula *H*′ = -*p*_i_ln*p*_i_. We calculated standard genetic parameters, including the average effective number of alleles (*N*_e_), Nei’s gene diversity (*h*)[49], Shannon’s information index (*I*)[46]and Nei’s unbiased measures of genetic distance [49] and gene flow [50],using the software program POPGENE version 1.31 [51].We also estimated the expected heterozygosity(*H*_e_), observed heterozygosity(*H*_o_), inbreeding coefficient (*F*_is_)and linkage disequilibrium across sampled loci and performed an analysis of molecular variance (AMOVA) to examine the partitioning of genetic variability within and among populations using the Arlequin software (version 3.5)[52]. The unweighted pair group method with arithmetic mean (UPGMA)dendrogram based on the genetic distances[53] was constructed using the software program TFPGA(version1.3). The principal component analysis of pairwise genetic distance between individuals was performed using GenAlEx version 6.4 [54].

We calculated the Bayesian estimate of panmictic heterozygosity (*h*_s_)as a measure of genetic diversity and *θ* ^*I*^(the Bayesian analog of Wright’s pairwise population distribution, *F*_st_) and *θ* ^B^(the Bayesian analog of Nei’s *G*_st_) as measures of population differentiation using the Hickory program (version 1.0)[55], which uses Markov chain Monte Carlo simulations to produce posterior distributions based on the data[55,56,57,58].Several runs of the analyses were performed with default sampling parameters (burn in = 50,000; sample = 250,000; thin = 50) to ensure consistency of results [59].The gene flow (*N*_m_) was estimated using the formula *N*_m_ = (1—*F*_st_) / 4*F*_st_[60].Bayesian model-based clustering analysis was used for determining the optimal number of genetic clusters among populations using the software program STRUCTURE 2.3.3 [61], which partitions individuals into numbers of clusters (*K*) based on the multi-locus genotypic data. The admixture model and correlated allele frequencies were used for each run with a burn-in period of 10,000 and 100,000 Markov chain Monte Carlo replications. The optimal *K* value, which indicates the number of genetically distinct clusters in the data, was determined from 10 replicate runs for each value of *K*[62]. The value of Δ*K* was based on the change in the log probability of the data between successive *K* values. Structure Harvester version 6.0 [63] was used to calculate parameters described by Evanno et al. [62].We confirmed that five independent runs consistently produced the same values.

We performed the Mantel test [64] in the Isolation By Distance program version 1.52 [65]to examine correlations of pairwise genetic distances of populations [49] and *F*_st_ with a geographic distance matrix.

## Results

### ISSR polymorphism

The 10 selected primers generated a total of 180 unambiguous and reproducible bands with sizes ranging from 220 to 2000 bp, of which 176 (97.7%) were polymorphic. The number of bands per primer ranged between 10 (primer 3A53) and 22 (primer 3A21), with an average of 18 bands per primer (Table 2). Among the 32,400 possible combinations, only 745 pairs (2.3%) showed significant linkage disequilibrium between loci (P ≤0.05). This is within the range expected by random chance, and therefore all 180 bands were considered independent loci. To determine PIC values for each ISSR primer, we analyzed the mean PIC values for all loci of each ISSR primer. We detected a high mean PIC value for ISSR primer3A39 (0.2783) and a low mean PIC value for ISSR primer 3A42 (0.1597) (Table 2). The average PIC per primer was 0.2155. The highest MI was observed with ISSR primer 3A39 (5.566) and the lowest with ISSR primer 3A53 (2.0988). There was a positive correlation between the MI and PIC values (*r* ^2^ = 0.909, P<0.05).The highest *H′* was observed with primer 3A07(0.3858) and the lowest with 3A42 (0.2779).

### Genetic variability within populations

The average observed number of alleles per population ranged from 1.672 in YMF to 1.811 in TF. FHB,WZT and SDG showed relatively high levels of observed allelic diversity, whereas populations SEG, XV and SP showed low levels of observed allelic diversity(Table 3). The mean effective number of alleles per population ranged from 1.2047 in WZT to 1.3211 in FHB. The YMF and XV populations showed relatively high levels of effective alleles, and the SEG and SP populations showed relatively low levels of effective allelic diversity. A similar pattern was observed in *h* and *I*, with the highest values of *h*(0.2018) and *I*(0.3180) in FHB and lowest values (*h* = 0.1419 and *I* = 0.2413) in WZT. The SDG, XV and YMF populations showed moderate values of *h* and *I*, and the values were lower for the TF,SEG and SP populations. The overall average values of *h* and *I* were 0.1989 and 0.3339, respectively. The values of *H*_e_ ranged from 0.2850 in WZT to 0.3473 in FHB. The YMF, XV and SDG populations showed relatively high values of *H*_e_, TF showed moderate values and SP and SEG showed relatively low values. The highest value of *H*_o_ occurred in the FHB population, followed by YMF and TF. The lowest value of *H*_o_ was found in SEG. The Bayesian estimates of the panmictic heterozygosity[32]ranged from 0.2192 in SP to 0.2648 in FHB. Overall, all measures of genetic diversity revealed the highest diversity in FHB, whereas SDG,XV,YMF and TF showed moderate levels of genetic diversity, and SEG,WZT and SP showed low levels of genetic diversity.

**Table 3:** Measures of genetic diversity and inbreeding coefficients in 8 populations of *L*. *regale*.

Population	Mean observed number of alleles	Mean effective nunumber of alleles	Nei’s gene diversities(*h*)	Shannon diversity index(*I*)	Average panmictic heterozygosity (*h*_s_) (*s*.*d*.)	Expected heterozygosity (*H*_e_)	Observed heterozygosity(*H*_o_)	Inbreedingcoefficient (*F*_is_) (*P*)
YMF	1.672 2	1.270 6	0.172 2	0.273 1	0.232 1 (0.005 5)	0.335 6	0.292 6	0.134 5(0.000 9)
FHB	1.772 2	1.321 1	0.201 8	0.318 0	0.264 8 (0.005 1)	0.347 3	0.294 0	0.160 1(0.000 0)
SDG	1.766 7	1.257 7	0.172 4	0.282 5	0.243 7 (0.005 8)	0.323 1	0.274 6	0.157 0(0.000 0)
SEG	1.755 6	1.230 5	0.153 2	0.253 4	0.223 1 (0.005 4)	0.293 2	0.247 8	0.161 3(0.000 0)
XV	1.738 9	1.266 3	0.174 5	0.282 1	0.242 8 (0.005 3)	0.328 4	0.278 9	0.157 0(0.000 0)
TF	1.811 1	1.234 3	0.162 7	0.274 0	0.244 2 (0.006 1)	0.306 8	0.280 0	0.091 7(0.011 7)
WZT	1.766 7	1.204 7	0.141 9	0.241 3	0.222 3(0.006 2)	0.285 0	0.275 3	0.036 1(0.180 8)
SP	1.705 6	1.229 5	0.152 9	0.251 0	0.219 2(0.005 4)	0.303 4	0.258 4	0.154 6(0.000 0)
Mean	1.748 6	1.251 8	0.166 4	0.271 9	0.239 2(0.005 5)	0.313 9	0.275 0	0.147 3(0.021 0)
Species	1.994 4	1.285 0	0.198 9	0.333 9	0.236 5(0.002 8)	0.335 6	0.292 7	0.135 3

The Hardy-Weinberg equilibrium test based on Markov chain Monte Carlo iterations revealed significant departures from Hardy-Weinberg equilibrium at five and six loci in populations XV and SEG, respectively. Similar departures from Hardy-Weinberg equilibrium were observed at one to three loci in the remaining populations, suggesting a varying degree of inbreeding or selection in populations.

### Genetic differentiation among populations

Overall, the population differentiation was moderate to high (*F*_st_ = 0.1909, P = 0.00). The results of the AMOVA indicated that most (80.91%) genetic variation was within populations, with a moderate amount (19.09%) among populations.

The inbreeding coefficient value (Table 4) was highest in the SEG population (0.1613) followed by FHB(0.1600) and SDG (0.1570). The inbreeding coefficient value of the WZT population was the lowest (0.0361). The average *θ* ^I^ was0.1897 (Model f = 0,s.d. = 0.0097) and the average *θ* ^B^ was 0.1849 (Full model, s.d. = 0.086). The *θ* ^B^ value was similar to, but slightly higher than, the *G*_st_value (0.1619) estimated using the POPGENE software, while *θ* ^I^ was similar to the *F*_st_ value (0.1909) calculated with the Arlequin software. The gene flow estimate (*N*_m_) was 1.0678 and suggests a moderate level of migration among populations. The pairwise Nei’s genetic distances (*D*_S_) among populations ranged from 0.0201 between SDG in the Minjiang River valley and TF in the Zagunao River valley to 0.0636 between the WZT and SP populations. The pairwise *F*_st_ values ranged from 0.1024 between SDG and TF to 0.2581 between XV and WZT (Table 5).

**Table 4:** The results of the Bayesian model based genetic differentiation and inbreeding coefficients in natural populations of *L*. *regale*.

^Model^	^Parameter^	^Mean^	^s.d.^	^2.5%^	^97.5%^	^DIC^
f free	θ^I^	0.256 6	0.020 6	0.216 9	0.296 8	5 683.38
	θ^B^	0.167 3	0.013 4	0.140 3	0.192 7	
θ ^ = 0^	θ^I^	0.169 5	0.015 2	0.141 9	0.201 3	9 379.62
^f = 0^	θ^I^	0.189 7	0.009 7	0.171 6	0.209 7	5 432.33
	θ^B^	0.117 6	0.006 1	0.106 2	0.129 7	
^Full model^	θ^I^	0.289 9	0.012 7	0.266 5	0.316 2	5 412.23
	θ^B^	0.184 9	0.008 6	0.168 2	0.202 3	

DIC: The Deviance Information Criterion. Models with the smaller DIC are preferred [55].

**Table 5:** Nei’s unbiased (1978) measures of genetic distance (below the diagonal) and Pairwise *F*_st_ (above the diagonal) observed in 8 populations of *L*. *regale*.

	YMF	FHB	SDG	SEG	XV	TF	WZT	SP
YMF		0.176 2	0.184 0	0.219 8	0.242 0	0.188 3	0.248 9	0.220 0
FHB	0.033 5		0.178 9	0.215 1	0.232 1	0.211 8	0.243 1	0.211 5
SDG	0.035 1	0.037 2		0.138 5	0.191 1	0.102 4	0.165 0	0.164 5
SEG	0.041 8	0.042 9	0.020 8		0.145 9	0.164 1	0.211 6	0.200 9
XV	0.057 4	0.055 3	0.032 1	0.038 8		0.178 7	0.258 1	0.263 6
TF	0.039 1	0.047 2	0.020 1	0.024 8	0.030 4		0.157 9	0.147 8
WZT	0.060 7	0.058 5	0.043 1	0.028 2	0.061 1	0.041 9		0.1943
SP	0.042 9	0.042 1	0.029 5	0.036 4	0.032 5	0.030 1	0.063 6	

There were no significant correlations between the geographic and genetic distances tested (*D*_S_: *r* = -0.2495, P ≤ 0.1130; *F*_st_: *r* = -0.2744, P ≤ 0.1040) or population differentiation parameters (*D*_S_: P ≤ -0.2841, *F*_st_:P≤ 0.8960).

**Fig 2:**
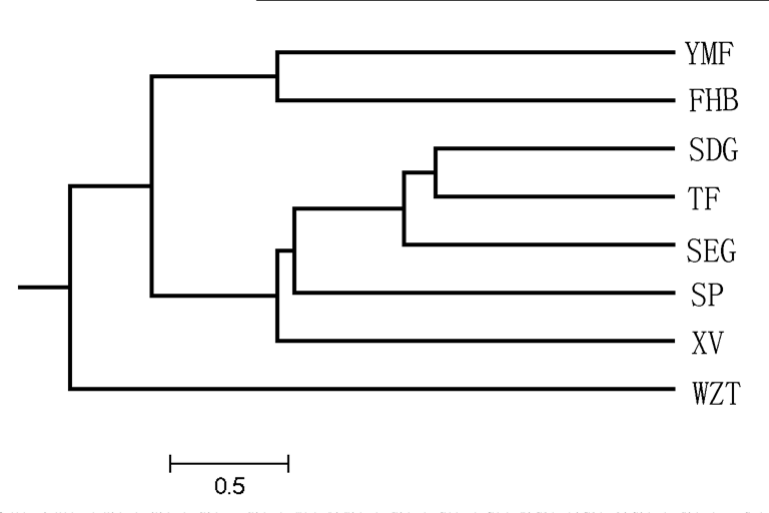
UPGMA dendrogram based on *D*_S_ values of eight *L*.*regale* populations.

The UPGMA clustering based on *D*_S_ values grouped populations into three distinct groups (Fig. 2). Group-A in the UPGMA tree consisted only of WZT. Group-B consisted of the SDG,SEG,XV,TF and SP populations, and Group-Cconsisted of YMF and FHB.

**Fig 3:**
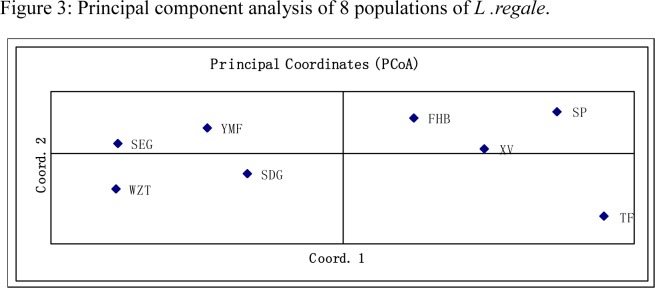
Principal component analysis of 8 populations of *L*. *regale*.

**Fig 4:**
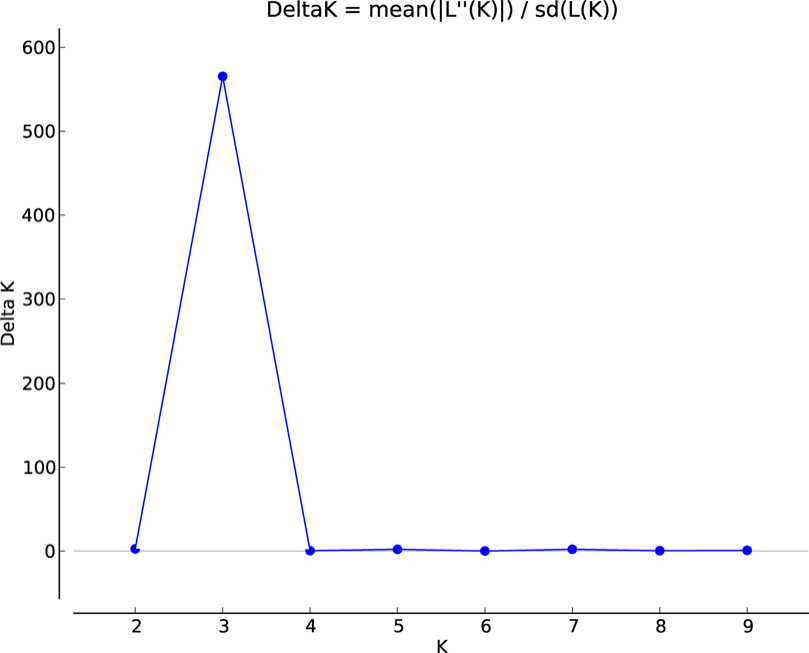
The relationship between Δ*K* and *K*as revealed by Structure Harvester. The highest peak was at *K* = 3.

**Fig 5:**
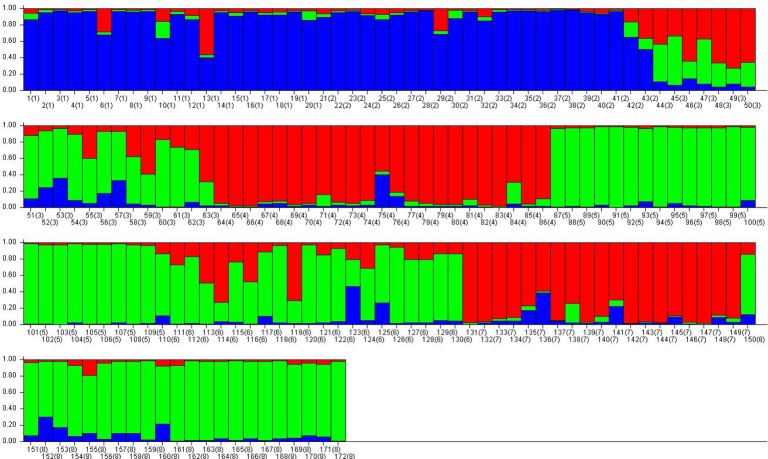
Bayesian model-based clustering (STRUCTURE)[60] of *L*. *regale* individuals (optimal value of *K* = 3). . YMF,2.FHB,3.SDG,4.SEG,5.XV,6.TF,7.WZT,8.SP. Blue: Group-I; red: Group-II; green: Group-III.

The percentages of variation explained by the first three axes in principal component analysis(PCA) were 58.37%,29.80% and10.02%.Theprincipal component analysis also clustered the eight populations into three distinct groups, but these were different from the three groups identified by UPGMA. In PCA, Group i consisted only of population TF, Group ii consisted of FHB, XV and SP, and Group iii consisted of YMF, SEG,SDG and WZT(Fig. 3).The Bayesian-based analysis of the population structure using the STRUCTURE software showed the highest log likelihood of change in *K* at three(Fig. 4), suggesting the existence of three genetically distinguishable groups. Individuals were then assigned to clusters based on posterior probability estimates of inclusion into each group. Individuals with a posterior probability ≥0.8 were considered confidently assigned to a single cluster. Those with split probabilities were assigned into two or more clusters and considered admixed individuals. The assignment of individuals into three clusters (Fig. 5) revealed that Group-I (blue) contained21.5% of the total individuals, with the majority being from the YMF and FHB populations. Another 20.3% of individuals, all but one (from SDG) of which were from the SEG and WZT populations, formed Group-II (red). Group-III (green) consisted of 30.8% of the individuals, the majority of which were from the XV and SP populations, with a few individuals from SDG and TF. The remaining 27.3% of individuals, mainly from the SDG and TF populations, showed high levels of mixed ancestry. Within this set, 46.8% of individuals clustered with all three groups, 29.8% clustered with Group-II and Group-III, 8.5% clustered with Group-I and Group-III, and 6.4% clustered with Group-I and Group-II. This indicates that the SEG and WZT populations, which are geographically separate, share the same ancestry. Similarly, the SDG and TF populations also showed mixed ancestry.

## Discussion

### ISSR Primers

The PIC of the ISSR primers ranged from 0.1597 to 0.2783, and the MI ranged from 2.0988 to 5.5660,which is lower than the SSR primers for this species[66].Therefore, the universal primers may not be particularly suitable to *L*.*regale*. Although the details of the mating system of *L*.*regale* are not well known, plants grown *ex situ*in conservation sites have been observed to produce seeds through self-pollination[67].The species is generally considered to have a mixed mating system, and reproduces both sexually and asexually through subterranean bulbs. The seeds of *L*. *regale* are winged, semi-circular, very thin and light (~5mg) [68]. Both pollen and seeds show adaptations for wind dispersal. The genetic diversity and population differentiation parameters which we obtained are in agreement with these features of *L*. *regale*.

### Genetic diversity

The evolutionary potential of a species largely depends on its level of genetic variability, and, for plants, the overall level of genetic variability in a population is often correlated with the geographical distribution range of the species[19,27,69].Compared with other narrowly distributed species of the genus *Lilium*, the genetic diversity of *L*. *regale*(*H*_o_ = 0.2750,*H*_e_ = 0.3139, *h* = 0.1989 and *I* = 0.3339) was moderately high. Higher genetic diversity in *L*. *regale* is likely to be attributable to its potential mixed mating system and long-range pollen and seed dispersal. The genetic diversity of *L*. *regale* is higher than the average genetic diversity reported for rare and common plant species at the population (rare: *H*_e_ = 0.113, *H*_o_ = 0.100; common: *H*_e_ = 0.150, *H*_o_ = 0.139) and species (rare: *H*_e_ = 0.142; common: *H*_e_ = 0.199) levels [70]. Overall, the genetic diversity measures that we found for *L*. *regale* were higher than those reported for narrowly endemic plant taxa [20,70,71].

### Mating system

The mating system can greatly influence genetic diversity both within and among populations. In general, most of the genetic diversity in self-pollinated plants is distributed among populations, whereas in outcrossed plants, most of the genetic diversity is distributed within populations[20]. The values for inbreeding and population genetic differentiation of *L*. *regale* observed in the present study (*F*_is_ = 0.1353, *F*_st_ = 0.1897, *G*_st_ = 0.1619) fall roughly in between those reported for selfed and outcrossed plants, further suggesting a mixed mating system in *L*. *regale*. Similarly, the self-pollination of *L*. *regale* [56] under *exsitu* conditions indicates that *L*. *regale* is self-compatible. However, the observed *G*_st_ value (0.1619) of *L*. *regale* was considerably lower than the *G*_st_ value (0.510) reported for generally self-pollinated plant species [20]. Furthermore, the strong fragrance of *L*.*regale* flowers suggests that they may attract animals as pollinators. In addition, strong and frequent wind in the Minjiang River valley [13]may facilitate pollination and seed dispersal. The trumpet-shaped flowers, large stamens, large numbers of highly viable pollen grains, and large, sticky stigma of *L*.*regale* also suggest wind pollination. Thus, it is possible that a combination of animal and wind pollination mechanisms function in *L*. *regale*. This is further supported by the fact that the *G*_st_ value of *L*.*regale* falls between those of typically wind-pollinated (*G*st = 0.100) and typically animal-pollinated (*G*_st_ = 0.216) plants [20].

Plants with narrow distribution ranges are prone to high levels of inbreeding, leading to loss of genetic variation and differentiation of populations[72,73,74]. Although *L*. *regale* is an endemic species with a limited distribution range, the levels of inbreeding in *L*. *regale* were lower than the values reported for rare (*F*_is_ = 0.175) and common (*F*_is_ = 0.184) plant species [70], and measures of genetic structure were also lower than those reported for rare (*F*_st_ = 0.212) and common (*F*_st_ = 0.198) species. The inbreeding value in *L*. *regale* was also lower than the range reported for the congeneric species *L*. *japonicum* Thunb. var. *abeanum*(Honda) Kitam (*F*_is_ = 0.042–0.385)[33]. Geneflow among populations decreases inbreeding, and the relatively high gene flow value of *L*. *regale* (*N*_m_ = 1.0678) that we observed may account for the low levels of inbreeding. The light, thin seeds of *L*. *regale* with wind velocity of 1.4–4m/s[75]may be dispersed to considerable distances [76]. However, it is unknown whether *L*. *regale* seeds and pollen commonly disperse across the rivers.

Although most species of the genus *Lilium* grow in the forest understory in shaded and moist conditions, *L*. *regale* grows in dry habitats in high sunlight in the Minjiang River valley. The high level of outcrossing due to wind-mediated pollination and seed dispersal may increase the genetic diversity in *L*.*regale*, allowing it to escape inbreeding depression and to respond to selection pressure under these harsh environmental conditions. Thus, the endemic and narrowly distributed *L*.*regale* maintains relatively high genetic diversity and gene flow through its adaptations for the environmental conditions in the Minjiang River valley.

### Genetic structure

The results of the AMOVA indicated that most of the variation in *L*.*regale* was within populations rather than among populations. The UPGMA, principal component and STRUCTURE analyses clustered the populations into three groups. However, the three groups determined by the three approaches consisted of different populations, with SEG,SDG,WZT and TF being the most differentially represented, which might be are result of the mixed ancestry of these populations. None of the clusters corresponded to the geographical distribution of populations, except for YMF and FHB.

The among-population genetic variation of *L*. *regale* was lower than the corresponding value of the self-incompatible species *L*. *brownii* (24.67%) [30]but was higher than the inter-population variation (5.09%) in the self-pollinating Taiwan lily, *L*. *longiflorum* Thunb. var. *formosanum* Baker [29],again suggesting a mixed mating system in *L*. *regale*.

### Asexual and sexual reproduction

Typically, clonal propagation is expected to give rise to higher levels of linkage disequilibrium, differentiation between populations, and heterozygosity than Hardy-Weinberg expectations for sexual reproduction [77,78]. The *L*.*regale* populations showed approximately2.3% linkage disequilibrium, and several of the populations showed significant departure from Hardy-Weinberg predictions, suggesting some clonal reproduction. The high number of ramets per genet will result in a large numbers of viable pollen and seeds, increasing the chances of successful establishment and survival of progeny derived from that genet. Moreover, for species with both asexual and sexual reproduction, efficient clonal propagation may result in a reduction in sexual reproduction[79,80]. As a consequence, a mixture of both clonal spread and sexual reproduction may be beneficial in a heterogeneous environment [81,82]. Therefore, the combination of sexual and clonal reproduction may contribute to the maintenance of high genetic diversity in *L*. *regale*.

Clonal plants may improve fitness in stressful environments by increasing resource acquisition through the development of a large rooting system, which may involve the physical integration of ramets[83], and reduction in resource expenditure for seed production and a concomitant increase in the proportion allocated to vegetative propagation[84]. Under these circumstances, low genetic diversity is expected in stressful environments [85]. In contrast, clonal plants may also increase investment in seed production under unfavorable conditions to respond to harsh environments [86,87]. Thus, high genetic diversity as a result of repeated seedling recruitment through sexual reproduction can also be expected under stressful conditions [88]. However, we did not encounter high levels of clonal reproduction in *L*.*regale*, suggesting that the high genetic diversity in this species is a consequence of recruitment of individuals through sexual reproduction.

## Conclusions

Despite being an endemic plant species confined to a relatively small geographical region in southwest China, *L*. *regale* shows relatively high genetic diversity, mostly within populations, and low levels of inbreeding compared with other narrowly distributed endemic plants. Strong winds prevalent in the river valley and a possible mixed mating system in this species may play important roles in maintaining a high level of gene flow among populations through pollen and seed dispersal. The fact that there were three genetically distinct groups of populations of *L*.*regale* should be taken into consideration in planning conservation and management programs for this species.

### Conservation implications

*Lilium regale* may serve as an important germplasm for the improvement of many lily varieties, and conservation strategies to protect native populations and prevent genetic erosion are urgently needed. For example, because *L*.*regale* grows in relatively dry environments, it likely carries alleles that confer tolerance to drought conditions that are predicted to become widespread under climate change scenarios[89].

Several measures must be implemented for the conservation and protection of *L*. *regale* populations. First, measures should be taken to minimize anthropogenic impact on native populations of *L*. *regale*. The natural habitat of *L*. *regale* is subject to heavy grazing and farming activities. The thin, dry soil of the habitat is prone to erosion leading to the loss of many populations and reducing the sizes of remaining populations[89]. Second, conservation plans should be implemented to preserve the maximum amount of genetic diversity [90]. We recommend the protection of large populations representing the three genetically distinct groups, such as the FHB, SEG and XV populations. Because physical barriers such as topographically dissected terrain and water bodies may hinder gene flow, several populations strategically located to promote gene flow should also be maintained. For instance, the location of the FHB population is central to maintaining gene flow across the river to increase network connectivity [76]. Third, genetic diversity parameters should be routinely monitored to capture long-term changes in the genetic diversity and implement mitigation measures in a timely fashion. Thus, the results of the present study may serve as the baseline data for future monitoring purposes. Finally, conservation of *L*. *regale* should focus on maintaining local adaptation. Thus, populations representing diverse habitats that include altitudinal or soil moisture extremes should be conserved.

Further analyses using more robust and more variable markers such as microsatellites or whole genome sequence-based single-nucleotide polymorphisms should be conducted to estimate the levels of self-pollination, consanguineous mating and outcrossing, and genome-wide associations with specific traits of ecological significance. Mating system studies combined with analyses of seed dispersal, germination and viability are needed to test some of the hypotheses resulting from the present study. Capitalizing on recent advances in conservation genomics techniques, including sequencing of whole genomes [91], many population genetic parameters such as genetic diversity, introgression and gene flow need to be studied at a higher precision for effective planning and implementation of conservation programs.
